# Genomic testing to determine drug response: measuring preferences of the public and patients using Discrete Choice Experiment (DCE)

**DOI:** 10.1186/1472-6963-13-454

**Published:** 2013-10-31

**Authors:** Mehdi Najafzadeh, Karissa M Johnston, Stuart J Peacock, Joseph M Connors, Marco A Marra, Larry D Lynd, Carlo A Marra

**Affiliations:** 1 Department of Medicine, Harvard Medical School, Boston, MA, USA; 2 Faculty of Pharmaceutical Sciences, University of British Columbia, Vancouver, BC, Canada; 3 British Columbia Cancer Agency, Vancouver, BC, Canada; 4 Genome Sciences Centre, Vancouver, BC, Canada; 5 Centre for Health Evaluation and Outcome Sciences (CHEOS), St. Paul’s Hospital, 1081 Burrard Street, Vancouver, BC, Canada; 6 Faculty of Medicine, University of British Columbia, Vancouver, Canada

**Keywords:** Pharmacogenomics, Genomic medicine, Personalized medicine, Genetic testing, Discrete choice experiment, Conjoint analysis, Preference elicitation, Cancer treatment

## Abstract

**Background:**

The extent to which a genomic test will be used in practice is affected by factors such as ability of the test to correctly predict response to treatment (i.e. sensitivity and specificity of the test), invasiveness of the testing procedure, test cost, and the probability and severity of side effects associated with treatment.

**Methods:**

Using discrete choice experimentation (DCE), we elicited preferences of the public (Sample 1, N = 533 and Sample 2, N = 525) and cancer patients (Sample 3, N = 38) for different attributes of a hypothetical genomic test for guiding cancer treatment. Samples 1 and 3 considered the test/treatment in the context of an aggressive curable cancer (scenario A) while the scenario for sample 2 was based on a non-aggressive incurable cancer (scenario B).

**Results:**

In aggressive curable cancer (scenario A), everything else being equal, the odds ratio (OR) of choosing a test with 95% sensitivity was 1.41 (versus a test with 50% sensitivity) and willingness to pay (WTP) was $1331, on average, for this amount of improvement in test sensitivity. In this scenario, the OR of choosing a test with 95% specificity was 1.24 times that of a test with 50% specificity (WTP = $827). In non-aggressive incurable cancer (scenario B), the OR of choosing a test with 95% sensitivity was 1.65 (WTP = $1344), and the OR of choosing a test with 95% specificity was 1.50 (WTP = $1080). Reducing severity of treatment side effects from severe to mild was associated with large ORs in both scenarios (OR = 2.10 and 2.24 in scenario A and B, respectively). In contrast, patients had a very large preference for 95% sensitivity of the test (OR = 5.23).

**Conclusion:**

The type and prognosis of cancer affected preferences for genomically-guided treatment. In aggressive curable cancer, individuals emphasized more on the sensitivity rather than the specificity of the test. In contrast, for a non-aggressive incurable cancer, individuals put similar emphasis on sensitivity and specificity of the test. While the public expressed strong preference toward lowering severity of side effects, improving sensitivity of the test had by far the largest influence on patients’ decision to use genomic testing.

## Background

Treatment options for cancer are mainly chosen based on the classification of the tumor and are usually based on the best knowledge of histogenesis, histological type, and stage of disease [1]. However, these criteria often fail to accurately differentiate among distinct subtypes of tumors, especially with respect to likelihood of response to treatment, forcing clinicians and patients to choose empirically. Thus, many patients end up experiencing significant side effects of chemotherapy without receiving clinical benefit [2].

Recent advances in genomics have created hope that genomic testing may help to identify patients who will likely respond to a particular drug and/or experience side effects. This information is valuable both for patients and physicians when choosing among possible treatment options and trading off between risks and benefits. For example, panitumumab, a drug for the treatment of colon cancer, was initially shown to be effective only in 10% of cases. However, genomic testing revealed that response rates were much higher in those without a KRAS mutation in their tumor [3]. Other examples are HER2 expression in breast cancer patients, which predicts response to trastuzumab [4] and the BCR-ABL genotype in chronic myeloid leukemia, which predicts response to imatinib mesylate [5].

Despite some clear advantages for the use of genomic tests to predict response to therapy, there are also some limitations. Genomic test results often have a probabilistic relationship with drug response – a certain genotype in the tumor may increase (or decrease) the probability of treatment response but this relationship is rarely absolute [3]. This prediction error in genomic testing may lead to the misclassification of those that will respond (i.e. sensitivity and specificity of tests are not perfect). In practice, the extent that an imperfect genomic test will be used is affected by multiple factors. Patients and physicians consider various factors such as invasiveness of the testing procedure, probability and severity of associated side effects of the treatment, and the overall costs before deciding about the usefulness of a genomic test [6,7].

The other important challenge is the impact of genomic testing on health care costs. An increasing number of diagnostic and predictive tests as a result of advances in genomics are creating increasing pressure on already soaring health care costs. There are ongoing debates about added clinical and economic value of these new technologies and appropriate methods for measuring those potential benefits [8,9]. New genomic tests, even if proven to deliver clinical benefit, are rarely cost saving. Thus, the decision about their overall value should be made based on the appropriate balance between clinical benefits and the costs of these technologies. In this context, it is important to determine which attributes of a genomic test are of more importance for patients when deciding about their treatment options. In general, approval and use of genomic tests varies widely across different jurisdictions and for different populations. Publicly (or privately) funded health care benefit providers are often interested in learning about tax payers’ (or privately insured populations’) opinion about the value of these genomic tests. Knowledge about these preferences will enable health benefit providers to select genomic tests with the highest perceived value when making funding decisions. This information can be used to prioritize future research areas and suggest aspects of genomic testing where improvement will have the most value to patients. Finally, this investigation may offer further insight about perceptions of patients who have directly experienced the disease and about their evaluation of different aspects of testing for cancer treatment. This information can potentially help physicians to offer treatment options that better match patients values and preferences [10].

Using a discrete choice experiment (DCE), we explored the relative impacts (i.e. relative preference weights) of different attributes of a genomic test on individuals’ decision to use the test for guiding cancer treatment. We investigated whether these relative impacts are influenced by type of cancer and its prognosis. Finally, we investigated how these relative impacts may differ between cancer patients and the public. Our knowledge about these relative preference weights can offer a value-based framework [11] for evaluating and comparing new genomic tests.

## Methods

### Study sample

Two samples from the public (sample 1 and sample 2) and a sample of current or former cancer patients participated in this study. The samples from the public (sample 1 and sample 2) were recruited by Ipsos Reid (Vancouver, British Columbia) and were representative of the Canadian general population in terms of demographics and socio-economic characteristics. The third sample (sample 3) consisted of current or former lymphoma patients who had voluntarily agreed be contacted about research projects in British Columbia (BC), Canada.

All subjects were invited to participate in this web-based study through email. All participants were at least 19 years old and were able to read and write in English. In the initial letter, we provided a brief description of the study and invited individuals to participate. Once they agreed, each participant provided informed consent and then followed a web link to the online questionnaire. Participants could choose not to answer any of the questions or withdraw at any point. The protocol for this study was reviewed and approved by the University of British Columbia - British Columbia Cancer Agency (BCCA) Research Ethics Board.

### Study procedure

At the beginning of the DCE questionnaire, we described one of two possible scenarios to the participants. We asked participants to imagine a situation where they have been diagnosed with either an aggressive curable cancer (scenario A) or a non-aggressive incurable cancer (scenario B) and that they have the option to choose a genomic test that can predict the likelihood of their response to a new chemotherapy (Table 1). We explained that the genomic test had limited accuracy, which might result in false negative (misclassifying responders as non-responders) and false positive (misclassifying non-responders as responders) predictions. Finally, we explained the attributes and levels in the DCE questionnaire (Table 2) and asked participants to complete 16 choice tasks [12,13]. We used the same choice questions for all three samples, but varied the underlying form of cancer described for one of the samples from the public: the preamble in the questionnaire described an aggressive curable cancer (scenario A) to participants in the first sample from the public and the sample from patients, and a non-aggressive, incurable cancer (scenario B) as the scenario for the second sample from the public. The design of the DCE questionnaire has been explained in the next section and a sample choice task has been presented in Table 3.

**Table 1:** Scenarios for DCE

**Scenario A:**	**Scenario B:**
**Aggressive curable Cancer**	**Non-aggressive incurable Cancer**
Imagine that you have recently been diagnosed with a fast-acting but curable form of cancer. Currently, approximately 50 out of 100 (50%) of patients are cured after the first round of chemotherapy. If you are cured by this initial treatment, you will have a normal life expectancy; otherwise your life expectancy is approximately 1 year. In this case you will be given the second round of chemotherapy but your chance of being cured is about 10 out of 100 (10%).	Imagine that you have recently been diagnosed with a slow-acting but incurable form of cancer. This means that the spread of the disease is usually slow, but treatments are only able to slow the spread further, and cannot cure the disease. Your life expectancy after being diagnosed with this type of cancer is approximately 10 to 13 years. You will receive treatment after you start experiencing symptoms, which may take several years after your initial diagnosis. Even if your treatment is successful, you are likely to experience numerous relapses, in which the disease returns after a period of improvement. These relapses will be treated until all options for treatment have been exhausted.
By adding a new medication to the first round of chemotherapy the cure rate increases from 50 out of 100 (50%) to 75 out of 100 (75%) . However, only some of individuals can benefit from the new medication (responders) and other individuals receive absolutely no benefit from adding the new medication to the standard chemotherapy (non-responders).	By adding a new medication to the first round of chemotherapy your life expectancy can be increased by 2 years on average. However, only some of individuals can benefit from the new medication (responders) and other individuals receive absolutely no benefit from adding the new medication to the standard chemotherapy (non-responders).
The downside of adding the new medication to the standard chemotherapy is that it increases the likelihood and severity of treatment side-effects.	The downside of adding the new medication to the standard chemotherapy is that it increases the likelihood and severity of treatment side-effects.

**Table 2:** Attribute and levels included in the DCE questionnaire

**Attribute**	**Levels**
**Untreated responders**^&^:	**5%, 20%, 35%, 50%**
Proportion of patients who could be cured by the new medication (responders) but will not receive it as a result of inaccurate genetic test result.	
**Unnecessary treatment of non-responders**^†^:	**5%, 20%, 35%, 50%**
Proportion of patients who would not benefit from the new medication (non-responders) but will receive it as a result of wrong genetic test result.	
**Severity of side effects:**	**Severe, Moderate, Mild**
The new medication may be associated with side effects such as nausea, hair loss, skin rash and fatigue. The potential levels of Side Effect Severity were:	
**Likelihood of side effects:**	**5%, 50%, 95%**
The side effects described in Attribute 3 will not necessarily occur for all individuals. Instead, they will occur with a particular percentage chance. Possible levels were:	
**Genetic test turnaround time:**	**2 days, 7 days, 12 days**
The time required to obtain the genetic test results, after the test has been performed.	
**Genetic test procedure:**	**Mouth swab, Blood sample, Tumor biopsy, Bone marrow biopsy, Liver biopsy**
Type of the procedure that is needed for doing the genetic test.	
**Genetic test cost:**	**$50, $500, $1000, $1500**
Please assume that you would be paying only for the genetic test out-of-pocket.	

^&^1-Sensitivity.

^†^1-specificity.

**Table 3:** A sample choice task

** Attributes**	**Option 1**	**Option 2**	**Neither**
Untreated responders	50 out of 100	5 out of 100	0 out of 100
Unnecessary treatment of non-responders	50 out of 100	5 out of 100	100 out of 100
Severity of side effects	Moderate	Mild	Severe
Likelihood of side effects	50 out of 100	5 out of 100	95 out of 100
Cost of genetic test	$1000	$500	$0
Genetic test turnaround time	7 days	2 days	0 days
Genetic test procedure	Bone Marrow Biopsy	Blood Sample	None
Which option you would choose?	O	O	O

The extent to which a genomic test will be used in practice is affected by the perceived benefits, risks and costs of using the genomic test. As such, in the DCE questionnaire participants needed to make a trade-off between the consequences of not taking the new chemotherapy when in fact it was beneficial, experiencing additional side effects of new chemotherapy without receiving any clinical benefit, the invasiveness of the genomic testing procedure, the test turnaround time, and the cost of the genomic test.

The descriptions at the beginning of the questionnaire explicitly stated that in the absence of a genomic test, all patients would be offered the new chemotherapy. As such, choosing the “neither” option in a choice task implied a respondent’s preference for opting-out from genomic testing and taking the new chemotherapy regardless of the likelihood of response. We did not specify the type of cancer, treatment, and the associated genomic test to increase the generalizability of the results. Nonetheless, the sample of patients in this study were former and current lymphoma patients in British Columbia, and the disease descriptions provided in the DCE questionnaires were similar to aggressive and non-aggressive types of lymphoma.

### Questionnaire design

Discrete choice experiment is a method to elicit individuals’ strength of preferences for different aspects of a health intervention (or a product in general). The concept of DCE is based on Random Utility Theory and the assumptions that: 1) a health care intervention (or any product or service in general) can be characterized by several attributes; and 2) individuals choose among available health interventions (or products or services) by evaluating and comparing their attributes [12-14]. These attributes can describe health outcomes (e.g. test accuracy, likelihood or severity of treatment side effects) or intervention process (cost, test procedure, or turnaround time).

In this study, we assumed that a genomically-guided cancer treatment could be described by seven attributes (Table 2). Considering that a large number of (hypothetical) treatment options can be generated by using these attributes and all various combinations of their levels (i.e. full factorial design), we implemented a fractional factorial design where we selected 10 versions of the DCE questionnaire each consisting of only 16 choice tasks. Therefore, each respondent had to complete a randomly assigned version of the DCE questionnaire that contained 16 choice tasks. In each choice task, respondents had to choose between two treatment options and a neither option. A sample choice task has been presented in Table 3 and the complete DCE questionnaire can be found in Additional file 1. The efficiency of our fractional factorial design was assured using simulation of responses. We generated large number of possible designs and then selected the design that provided the most precise coefficient estimates (i.e. smallest standard errors) and a better D-efficiency given the sample size [14,15]. The statistical design of the questionnaire ensured that a random selection of responses would result in preference weights that are not statistically different from zero (i.e. non-informative coefficient estimates).

Several sources were used for selection of attributes including published literature, physicians’ opinion, and feedbacks that we received from three pilot surveys. We identified several studies that had investigated characteristics of pharmacogenomic testing and their impact on patients’ and physicians’ decisions for utilizing them [16-18]. We compiled a list of attributes based on the results of these studies and discussed this list with physicians who were in direct contact with cancer patients in the BC cancer agency. We then selected the seven attributes deemed to have the greatest influence on patient’s decisions about treatment options. These attributes and levels were then tested in a pilot study where 7 former cancer patients and 50 individuals from the public completed the preliminary version of the DCE questionnaire. By analyzing the data in the pilot phase, we examined rationality and consistency of the responses and whether the estimated coefficients conformed to our prior expectation in terms of direction and sign. Our prior expectation was based on the assumption that individuals’ preferences (and willingness to pay) decrease by decreasing sensitivity and specificity of the test, and by increasing severity and likelihood of side effects, turnaround time, cost, or invasiveness of the testing procedure. Using this approach, we ensured that the respondents understood the content of the DCE questionnaire and our instructions for completion of choice tasks. Furthermore, we used the comments provided by respondents at the end of the questionnaires to hone the preamble, descriptions, attributes, and levels used in the final version of the questionnaire.

Two out of 16 choice tasks in the DCE questionnaire contained a clearly dominant option. By checking answers to these fixed choice tasks, we tested if respondents actually read and understood the DCE questionnaire. These fixed choice tasks are usually part of the DCE questionnaire design in order to verify consistency and rationality of responses. We also included the “neither” option in the choice tasks to provide the possibility to opt-out whenever none of the presented alternatives was adequately attractive to the respondent. Thus, we avoided forcing non-demanders to choose an alternative and ensured estimation of unconditional rather than conditional preferences [14]. The design of the web-based questionnaire, which facilitated direct data entry into our secured server, was done using the Choice Based Conjoint (CBC) application of Sawtooth (Sawtooth software Inc, SSI web version 6.6.6).

### Statistical analysis

Assuming the general framework used in random utility theory [14], given a set of options, the log odds ratio of choosing one of the options is proportional to a linear function of attributes of that option. Therefore, by gathering stated choice data using a DCE questionnaire with a known statistical design and by knowing attributes and levels presented in each choice task, the coefficients of attributes can be estimated using generalized linear models. These coefficients, also known as relative preference weights, reflect average impact of attribute levels on likelihood of being chosen as the preferred option. Also the ratio of coefficients can be interpreted as marginal rate of substitution (MRS) between any two attributes. By inclusion of cost as an attribute in the DCE questionnaire, the marginal rates of substitution between each attribute and cost, also known as Willingness to Pay (WTP) [14], can be calculated. WTP can provide useful interpretations for estimated preference weights as they indicate how much individuals on average are willing to pay to receive a certain amount of change in one of the attribute levels [14]. The odds ratios (OR) associated with each attribute levels also were calculated. These odds ratios suggest, given two options with the same attribute levels, how a change in one of the attribute levels will affect the odds of becoming the preferred choice.

The choice data were effect-coded for attributes with discrete values, with the exception of cost, which was modeled as a continuous variable [19]. Effect coding of choice data, instead of continuous coding, relaxes linearity assumptions and allows detecting non-linearity of preference weights in regards to different levels of an attribute. Also modeling cost as a continuous variable allowed us to estimate WTP values in a way that is easy to interpret. An alternative specific variable was dummy coded and indicated the situations where “neither” was chosen [14,20]. The choice data was analyzed using PROC MDC, SAS 9.2. We pooled the choice data from two samples from the public who completed the questionnaire under scenario A and scenario B and estimated a conditional logit model using choice as the dependent variable. We defined a dummy variable that indicated the scenario in the pooled data. By including interaction terms between this dummy variable and attribute levels in the regression analysis, we compared the estimated preference weights across two samples from the public. We also used the same approach to compare estimated preference weights in the samples from the public and patients who had both completed the questionnaire under scenario A. However, prior to doing this analysis, we used the propensity score method to select a subsample of the public that were similar to the sample of patients in terms of age, education, income, and having dependent children. Considering that the characteristics of patients in our sample were different from the public, using propensity scores was necessary to increase comparability of the results across the samples from the public and patients in our analysis.

There are a variety of statistical methods for the analyses of DCE data that range from conditional logit models to Bayesian mixed logit models [21] and Latent Class Analysis (LCA) [22]. Critical assessment of these methods can be found elsewhere [23]. We chose conditional logit model for analyses of the DCE data in this study. However, we verified the estimated results and robustness of our findings by re-running the regressions using a mixed logit model.

## Results

### Sample characteristics

Invitations were initially sent to 904 and 836 individuals from the public for participation in the study under scenarios A and B, respectively. Although 588 (65%) individuals in scenario A and 578 (69%) individuals in scenario B provided their responses to the questionnaires, some of the questionnaires contained uncompleted choice tasks. To avoid potential bias as a result of imbalanced frequency of responses, we decided to restrict our analysis to the data from questionnaires with complete responses to all choice tasks (533 individuals in scenario A and 525 individuals in scenario B). Our sample of patients was limited to an email list provided by BC cancer Agency (BCCA). We initially contacted a list of 84 patients through email and 54 (64%) patients agreed to participate in this study. However, after excluding incomplete responses, we had choice data from 38 patients for the final analysis.

Table 4 has summarized the characteristics of the participants in the three samples. Mean age in the sample of patients was 58.2 years, about 10 years higher than in the samples from the public. Also 36.1% of individuals in the sample from patients reported a household income of ≥ Can $125,000 (This rate was 6.6% and 5.5% in the samples from the public). Patients who participated in this study were also highly educated and 32.4% had a master or doctorate degree (the proportions of individuals with master or doctorate degree were 2.5% and 4.1% in the samples from the public under scenario A and scenario B, respectively).

**Table 4:** Characteristics of participants

	**The public, scenario A**	**The public, scenario B**	**Patients, scenario A**
	**N = 533**	**N = 525**	**N = 38**
**Age (years)**	**N = 512**	**N = 510**	**N = 37**
Mean (std)	48.2 (15.7)	47.6 (15.9)	58.2 (9.4)
**Education (%)**	**N = 529**	**N = 514**	**N = 37**
Some high school	6.8%	6.6%	0%
High school	42.2%	45.5%	2.7%
College	36.1%	35.4%	27.0%
Bachelor degree	12.5%	8.4%	37.8%
Master degree	1.9%	3.5%	27.0%
Doctorate	0.6%	0.6%	5.4%
**Gender**	**N = 522**	**N = 516**	**N = 36**
Female	48.5%	50.6%	58.3%
Male	51.5%	49.4%	41.7%
**Number of dependent children**	**N = 532**	**N = 517**	**N = 36**
None	57.9%	56.5%	69.4%
1	14.3%	15.5%	5.6%
2	16.2%	16.1%	13.9%
3 or more	11.6%	12.0%	11.1%
**Description of current health situation**	**N = 526**	**N = 521**	**N = 37**
Excellent	8.9%	11.3%	10.8%
Very good	29.9%	28.6%	16.2%
Good	29.1%	33.6%	32.4%
With some health problems	28.7%	23.8%	21.6%
Having serious health problems	3.4%	2.7%	18.9%
**If knew anyone diagnosed with cancer**	**N = 528**	**N = 519**	**N = 36**
Yes, very closely	17.8%	16.2%	2.8%
Yes	38.1%	41.6%	30.6%
No	44.1%	42.2%	66.7%
**Household’s annual income (Can$)**	**N = 516**	**N = 509**	**N = 36**
Less than 25000	15.5%	13.2%	2.8%
25000 to 50000	30.2%	35.6%	27.8%
50000 to 75000	23.1%	23.6%	11.1%
75000 to 100000	16.9%	12.6%	13.9%
100000 to 125000	7.8%	9.6%	8.3%
More than 125000	6.6%	5.5%	36.1%

### Estimation results

#### Comparing preferences of the public under two scenarios A and B

The estimated preference weights, odds ratios, and the WTP associated with the levels in each attribute have been reported in Table 5.

**Table 5:** Estimated preference weights and Willingness to Pay (WTP) in samples from the public

	**The public, scenario A**	**(N = 533)**		**The public, scenario B**	**(N = 525)**		**P-value for difference**
	**Coefficient (s.e.)**	**OR**	**MWTP**	**Coefficient (s.e.)**	**OR**	**MWTP**	
**Untreated responders**^&^							
5%	0.1748 (0.0266)**	1.41	1,331	0.2577 (0.0270)**	1.65	1,344	0.0241
20%	0.0367 (0.0265)	1.23	796	0.0324 (0.0274)	1.32	740	0.8487
35%	−0.0429 (0.0276)	1.13	487	−0.0465 (0.0285)	1.22	528	0.9315
50%	−0.1686 (0.0466)	1	Ref	−0.2436 (0.0479)**	1	Ref	
**Unnecessary treatment of non-responders**^†^							
5%	0.1008 (0.0268)**	1.24	827	0.2452 (0.0270)**	1.50	1,080	0.0004
20%	0.0065 (0.0274)	1.13	461	0.0377 (0.0288)	1.22	524	0.6691
35%	0.0051 (0.0272)	1.12	456	−0.1251 (0.0281)**	1.03	88	0.0115
50%	−0.1124 (0.0470)	1	Ref	−0.1578 (0.0485)**	1	Ref	
**Severity of side effects**							
Mild	0.3319 (0.0205)**	2.10	2,882	0.3295 (0.0211)**	2.24	2,165	0.3838
Moderate	0.0798 (0.0210)**	1.63	1,905	0.1484 (0.0217)**	1.87	1,679	0.0621
Severe	−0.4117 (0.0293)**	1	Ref	−0.4779 (0.0303)**	1	Ref	
**Likelihood of side effects**							
5%	0.2490 (0.0204)**	1.62	1,861	0.2622 (0.0213)**	1.75	1,497	0.2245
50%	−0.0179 (0.0209)	1.24	826	0.0340 (0.0214)	1.39	885	0.2261
95%	−0.2311 (0.0292)**	1	Ref	−0.2962 (0.0302)**	1	Ref	
**Genetic test turnaround time**							
2 days	0.1213 (0.0210)**	1.26	911	0.1266 (0.213)**	1.27	650	0.4533
7 days	−0.0076 (0.0208)	1.11	411	−0.0107 (0.0218)	1.11	282	0.5396
12 days	−0.1137 (0.0296)**	1	Ref	−0.1159 (0.0305)**	1	Ref	
**Genetic test procedure**							
Mouth swab	0.2962 (0.0304)**	1.75	2,162	0.3045 (0.0311)**	1.73	1,474	0.9942
Blood sample	0.2863 (0.0320)**	1.73	2,124	0.3882 (0.0332)**	1.88	1,698	0.5738
Tumor biopsy	−0.0416 (0.0326)	1.25	853	−0.0809 (0.0332)**	1.18	440	0.8152
Bone marrow biopsy	−0.2792 (0.0321)**	0.98	−68	−0.3666 (0.0339)**	0.89	−325	0.6879
Liver biopsy	−0.2617 (0.0636)**	1	Ref	−0.2452 (0.0675)**	1	Ref	
**Neither (No test)**	−0.6323 (0.0379)**	0.53	−2,451	−0.4967 (0.0389)**	0.61	−1,332	0.0169
**Genetic test cost**	−0.00026 (0.00003)**		Ref	−0.00037 (0.00003)**		Ref	0.0091
McFadden’s LRI	0.17						
Adjusted Estrella	0.33						
Log-likelihood Ratio	6216.5						
AIC	31050						
Schwartz Criterion	31329						

** p-value < 0.01.

^&^1-Sensitivity.

^†^1-specificity.

The results suggested that in aggressive curable cancer (scenario A), the preference weight of the public for “sensitivity: 50%” was −0.1686 (s.e. 0.466) and it increased to 0.1748 (s.e. 0.0266) for a test with “sensitivity: 95%” (Table 5). Alternatively, the impact of test sensitivity on respondent’s choice is evident in the reported ORs and WTPs. For example, everything else being equal, the odds of choosing a test with 95% sensitivity were 1.41 times the odds of choosing a test with 50% sensitivity and they were willing to pay $1331 for increasing test sensitivity from 50% to 95%. However, they were willing to pay only $796 and $487 for increasing sensitivity to 80% and 65%, respectively. In non-aggressive incurable cancer (scenario B), preference weights of “sensitivity: 95%” and “sensitivity: 50%” were 0.2577 (s.e. 0.270) and −0.2436 (s.e. 0.0479), respectively. Increasing sensitivity from 50% to 95% increased the odds of choice by 1.65 times. Although this preference weight in scenario B was larger compared to scenario A (0.2577 vs. 0.1748, difference p-value = 0.0241), corresponding willingness to pay values were comparable ($1331 vs. $1344 in scenario B and A, respectively). Preference weights and WTPs for a test with sensitivity of 80% or 65% in scenario B were not significantly different from corresponding values in scenario A.

In scenario A, the odds of choosing a test with 95% specificity were 1.24 times the odds of choosing a test with 50% specificity and the public was willing to pay $827 for this amount of improvement in specificity level. The preference weight for 95% specificity was more than two-fold larger under scenario B compared to scenario A (0.2452, 0.1008, difference p-value < 0.001). Therefore, under scenario B, the odds of choosing a test with 95% specificity were 1.50 times the odds of choosing a test with 50% specificity and the corresponding WTP was $1080. Also in scenario B, the preference weight of 65% specificity was negative (−0.1251) and statistically different (difference p-value = 0.0115) from its counterpart under scenario A (0.0051). The public perceived little value in increasing specificity from 50% to 65% in scenario B.

Reducing severity of treatment side effects from severe to mild was associated with large ORs in both scenarios (OR = 2.10 and 2.24 in scenario A and B, respectively). The public was willing to pay as much as $2882 and $2165 to receive a treatment with mild rather than severe side effects in aggressive curable cancer (scenario A) and non-aggressive incurable cancer (scenario B), respectively. Furthermore, the odds of choosing a treatment with 5% likelihood of side effects were 1.62 and 1.75 times the odds of choosing a treatment with 95% likelihood of side effects in scenario A and B, respectively.

Shortening test turnaround time from 12 days to either 7 days or 2 days had the smallest impact on preference weights, ORs, and WTPs under both scenarios. In contrast, the level of invasiveness of the testing procedure had a large impact on estimated preference weights, ORs, and WTP values in both scenarios. For example, the public was willing to pay $2162 and $1474 for a genomic test that could be performed using a mouth swab rather than one involving a liver biopsy in scenario A and B, respectively.

Individuals from the public had negative preference weights for opting out from genetic testing (i.e. choosing “neither” option). The preference weight was a larger negative number under scenario A compared to scenario B (−0.6323 in scenario A vs. -0.4967 in scenario B, difference p-value = 0.0169). The ORs of opting-out from genetic testing (vs. taking a test) were 0.53 and 0.61 in scenario A and B, respectively. The public had a larger WTP for having a test in aggressive curable cancer scenario ($2451) compared with non-aggressive incurable cancer ($1332). Finally, the preference weight for “genetic test cost” was a larger negative number under scenario B compared to scenario A (−0.00026 in A vs. -0.00037 in B, difference p-value = 0.0091), indicating that the public was more sensitive to price in scenario B.

#### Comparing preferences of the public with preferences of patients under scenario A

Using propensity scoring we identified a subsample of the public (N = 83) who had similar characteristics to patients (N = 38) in terms of age, education, income, and number of dependent children. Next we pooled the data from two samples (N = 121) and fitted a conditional logit model to estimate preference weights, ORs, and WTPs associated with each attribute levels (Table 6).

**Table 6:** Estimated preference weights and Willingness to Pay (WTP) in a propensity score matched subset of the public and patients

	**The public, scenario A**		**(N = 83)**	**patients, scenario A**		**(N = 38)**	**P-value for difference**
	**Coefficient (s.e.)**	**OR**	**MWTP**	**Coefficient (s.e.)**	**OR**	**MWTP**	
**Untreated responders**^&^							
5%	0.248 (0.0687)**	1.58	2,658	0.8794 (0.1068)**	5.23	12,820	<.0001
20%	0.0528 (0.0676)	1.30	1517	0.0442 (0.1068)	2.27	6,346	0.6084
35%	−0.0942 (0.0702)	1.12	657	−0.1492 (0.1133)	1.87	4,847	0.6582
50%	−0.2066	1	Ref	−0.7744	1	Ref	
**Unnecessary treatment of non-responders**^†^							
5%	0.1867 (0.0679)**	1.39	1,919	0.1083 (0.1083)	1.59	3,580	0.9663
20%	0.0134 (0.0697)	1.17	906	0.2391 (0.1112)*	1.81	4,594	0.1598
35%	−0.0586 (0.0683)	1.09	485	0.0062 (0.1088)	1.43	2,789	0.657
50%	−0.1415	1	Ref	−0.3526	1	Ref	
**Severity of side effects**							
Mild	0.2712 (0.0524)**	2.10	4,327	0.3084 (0.0839)**	2.09	5,716	0.7
Moderate	0.1976 (0.053)**	1.95	3,897	0.1206 (0.0871)	1.73	4,260	0.3008
Severe	−0.4688	1	Ref	−0.4290	1	Ref	
**Likelihood of side effects**							
5%	0.2645 (0.0521)**	1.65	2,937	0.2735 (0.0838)*	1.60	3,650	0.8119
50%	−0.0267 (0.0529)	1.24	1,235	−0.0762 (0.0864)	1.13	939	0.5678
95%	−0.2378	1	Ref	−0.1973	1	Ref	
**Genetic test turnaround time**							
2 days	0.1673 (0.0531)**	1.40	1,978	0.0174 (0.087)	1.15	1,117	0.2467
7 days	0.0036 (0.0521)	1.19	1,021	0.1093 (0.0857)	1.27	1,829	0.5259
12 days	−0.1709	1	Ref	−0.1267	1	Ref	
**Genetic test procedure**							
Mouth swab	0.2591 (0.0783)**	1.75	3,258	0.3918 (0.1239)*	2.43	6,881	0.4303
Blood sample	0.3382 (0.0825)**	1.89	3,720	0.1168 (0.1322)	1.85	4,750	0.2508
Tumor biopsy	−0.04 (0.0828)	1.29	1,509	−0.0829 (0.1342)	1.51	3,202	0.7787
Bone marrow biopsy	−0.2593 (0.081)**	1.04	226	0.0702 (0.1286)	1.76	4,388	0.1121
Liver biopsy	−0.2980	1	Ref	−0.4959	1	Ref	
**Neither (No test)**	−1.0346 (0.1037)**	0.36	−6,050	−0.1185 (0.1382)	0.89	−919	0.0002
**Genetic test cost**	−0.00017 (0.00007)*		Ref	−0.00013 (0.00012)		Ref	0.1901
McFadden’s LRI	0.20						
Adjusted Estrella	0.35						
Log-likelihood Ratio	832.8						
AIC	3493						
Schwartz Criterion	3694						

** p-value < 0.01; * p-value < 0.05.

^&^1-Sensitivity.

^†^1-specificity.

The preference weight of patients for “sensitivity: 95%” was significantly larger compared to the public (0.2480 in the public vs. 0.8794 in patients, difference p-value < 0.001). This large difference in preference weights for “sensitivity: 95%” also translated into large differences in WTP estimates ($2,658 for the public vs. $12,820 for patients) and ORs (1.53 vs. 5.23, respectively). Patients’ had consistently larger preference weight for better sensitivity and specificity of the test, as was evident based on ORs and WTP values associated with different levels of sensitivity. Among patients, the odds of choosing a genetic test that requires “mouth swab” were 2.43 times the odds of a test that needs liver biopsy. Patients also preferred a test that involves “Bone marrow biopsy” instead of “liver biopsy” (OR = 1.76), while the public considered both types of biopsies equally unfavorable (OR = 1.04). There was a large difference between preference weight of the public versus patients for opting-out from the test (−1.0346 in the public vs. -0.1185 in patients, difference p-value = 0.0002). Consequently, the public was willing to pay as high as $6050 for having a genetic test while patients’ WTP for genetic testing was only $919. This indicated patients had significantly less aversion to opting out of genomic testing.

## Discussion

This study shows the relative impact of different properties of genomically-guided cancer treatment on test uptake. Change in severity and likelihood of side effects as well as the test procedure have the largest influence on the public’s decision to use genetic testing. In contrast, improving sensitivity of the test had a larger influence on patients’ decision to use genomic testing.

The type of cancer and its prognosis also influenced the preferences of the public for different attributes of genomic testing. When we compared the results in the two samples from the public, we found that in aggressive curable cancer, individuals emphasized the sensitivity rather than specificity of the test. In contrast, for a non-aggressive incurable cancer, individuals put similar emphasis on the sensitivity and specificity of the test and expressed strong (positive and negative) preferences toward (high and low) specificity of the test. Furthermore, under this scenario (non-aggressive incurable cancer) the public also had a larger negative preference toward the cost of genomic testing. Because for a non-aggressive incurable cancer the change in the survival is ultimately small and is expected to be materialized after 13 years, this lead to the public discounting the benefits of new chemotherapy and becoming more selective about accuracy of genomic testing in this scenario.

Our study suggests that patients and the public have different perceptions about the value of various aspects of genomic testing to guide cancer treatment when facing an aggressive curable cancer. Based on our results, patients were mostly concerned about improving sensitivity of the test (and presumably their survival chance), and in the absence of an adequately sensitive test they preferred opting-out from genomic testing and taking the treatment regardless of its side effects. Conversely, the public had a large negative preference weight for opting-out from genomic testing suggesting that they are more inclined to use a test even with inadequate accuracy. This information may help physicians to tailor their clinical advice considering type of cancer and previous experience of their patient with cancer treatment. For example, if the prognosis of disease is expected to be similar to our scenario for non-aggressive incurable cancer, then perhaps discussing false positive rates of available tests can be of great importance for the average patient. Also, the observed differences between preferences of patients and the public about different biopsy procedures suggest that perhaps physicians can help patients who have no prior experience of cancer treatment in developing a more realistic perception about the relative invasiveness of these procedures.

There is a paucity of studies about preferences for characteristics of genomic testing. The increasing number of new genomic tests ensuing from fast developments in genomic sciences underlines the need for further investigations in this area. Knowledge about strength of preferences toward different attributes of genomic testing can lead us toward value-based evaluation of these new technologies. In health care systems that rely on public funding resources, by considering these preference weights in funding decisions, genomic tests with potentially higher value for a covered population can be determined. In addition, physicians can have better understanding about patients’ priorities given the type and prognosis of the disease. The differences in preferences of patients and the public shown in our study also suggests areas that physicians should emphasize when communicating with recently diagnosed patients who presumably have no prior experience of the disease. In a study conducted by Griffith et al., willingness to pay for receiving breast cancer genomic services was estimated by conducting a DCE on 242 individuals with high, moderate, and low risk of developing breast cancer [24]. Using a DCE and following a rigorous methodology, Hall and colleagues [25] explored the factors that influenced participation in genomic carrier testing for Tay Sachs and cystic fibrosis among a sample from the general community and a sample of the Ashkenazi Jewish community. A recent study [26] also used DCE to estimate the tradeoffs among sensitivity, turnaround time, and cost of a postnatal genomic test to predict genomic abnormalities causing mental retardation in children. Finally, in a study done by Herbild et al. [27], they elicited preferences in the Danish general population for taking a pharmacogenomic test that could improve treatment of depression.

Patients’ emphasis on sensitivity also has been shown in the context of using usual screening tests for colorectal cancer [28]. In exploring preferences of 1047 patients with a history of colorectal cancer for different screening modalities, Marshall et al. used a DCE and estimated how likelihood of uptake may be affected by different characteristics of the test. Similar to our results, they found that sensitivity of the test has the largest impact on the likelihood of uptake among these patients. A cross sectional survey study by Haga et al. also showed that primary care physicians consider the severity of side effects followed by predictive accuracy of a phramacogenomic test as the factors that have the largest influence on their decision to prescribe it to their patients, while turnaround times have a smaller influence on their decision for using pharmacogenomics testing [16]. These results, when considered in the context of our findings, suggest that perhaps neither the public nor physicians share patients’ highest priority for better test sensitivity. Direct comparison of physicians and patients preferences about genomic testing can provide useful insight about this matter and should be pursued further in future research.

The distinct characteristic of our study is utilizing three samples to demonstrate how the type of cancer and its prognosis affected preferences for a genomic test, and how preferences of patients differed from those of the public. Also, in contrast with previous studies, the results of our study are applicable to most genomic tests for guiding cancer treatment, as we did not specify the type of cancer, treatment, or the associated genomic test. However, we acknowledge that in the absence of specifying the type of cancer, participants may make various assumptions about possible prognosis and potential outcomes. Therefore, this can be seen as a limitation of our study as well. Throughout this study, participants provided their choices considering the following assumptions: 1) if they decided to opt-out from genomic testing, they would receive the new treatment regardless of its effect, and 2) the new treatment was covered by their insurance policies. We acknowledge that under different circumstances in terms of the effect of genomic testing on access to the new treatment, the current results may not apply. The larger standard errors around the estimated coefficients in patients suggested that this sample was slightly underpowered. However, the sample size was restricted to a list of lymphoma patients in BC cancer agency’s contact list and willingness of those approached to participate and thus could not be increased. Despite this limitation, all of the point estimates in the sample of patients were in line with our prior expectations in terms of the order of their magnitudes and corresponding signs. Moreover, this sample was not an archetypal sample of cancer patients in BC, as they had high income, high education level, and were 10 years older on average. Therefore, we used propensity scoring to find a subsample of the public with similar characteristics to increase comparability of the results. This issue, however, potentially limits the external validity of the results based on these samples. Actual decisions that patients or the public make in real life situations may deviate from their stated preference in surveys like ours. This effect has been shown in the context of genetic testing as well [29]. However, several studies provide evidence suggesting strong correlation between stated and real WTP [30] and preferences [31]. Answering DCE questions can be a complex task and accuracy of responses may eventually depend on participants’ numeracy level (i.e. ability to interpret quantitative information) [32], language skills, familiarity of subject, and attentiveness while completing the questionnaire. We have used several standard approaches to assure quality of the data by including a fixed choice task to test rationality of responses and by checking the time that each respondent spent on completing the questionnaire. Overall, given the directions and signs of the estimated preference weights, we believe that our results are robust and have not been compromised by these potential problems. Finally, we acknowledge that the factors that can affect uptake of a genomic test are not limited to the seven attributes that we have included in the current DCE design. We excluded several important aspects (e.g. risk involved in testing procedure) that individuals may take into account when making their actual decision about using genomic testing. This selection was to use the minimum possible number of attributes and avoid overly complex choice tasks [33].

Our study demonstrates individuals’ preference strength toward characteristics of a genomic test when they are faced with an aggressive but curable cancer versus a non-aggressive and incurable cancer. Additionally, these results suggest which characteristics of genomic testing have a larger potential value for society and patients. Physicians may find these average preferences as a benchmark when providing treatment advice about pharmacogenomics testing to cancer patients. These preference weights also can be used to inform funding decisions by incorporating relevant populations’ valuation of different aspects of genomic testing.

## Conclusions

We explored the relative impact of different properties of genomically-guided cancer treatment on test uptake. We found that the type and prognosis of cancer affected preferences for genomically-guided treatment. Our results also suggest that patients and the public have different perceptions about the value of various aspects of genomic testing to guide cancer treatment. Physicians may find these average preferences as a benchmark when providing treatment advice about pharmacogenomics testing to cancer patients. These preference weights also can be used to inform funding decisions by considering relevant populations’ valuation of different aspects of genomic testing.

## Competing interests

Authors have no financial or non-financial competing interests in relation with the content of this study.

## Authors’ contributions

MN: Design, Data, Analysis, Interpretation, Drafting the Manuscript, and Final Approval; KJ: Design, Drafting the Manuscript, and Final Approval; SP: Data, Drafting the Manuscript, and Final Approval. JC: Conception, Data, Interpretation, Drafting the Manuscript, and Final Approval; MM: Conception, Drafting the Manuscript, and Final Approval; LL: Interpretation, Drafting the Manuscript, and Final Approval; CM: Conception, Design, Data, Interpretation, Drafting the Manuscript, and Final Approval. All authors read and approved the final manuscript.

## Pre-publication history

The pre-publication history for this paper can be accessed here:

http://www.biomedcentral.com/1472-6963/13/454/prepub

## Supplementary Material

Additional file 1 The DCE questionnaire. Click here for file
