# Supplementary material for: Genomic testing to determine drug response: measuring preferences of the public and patients using Discrete Choice Experiment (DCE)
Source: BMC Health Serv Res. 2013 Oct 31;13:454. doi: 10.1186/1472-6963-13-454 (PMC3827922; doi:10.1186/1472-6963-13-454)
Supplement: Additional file 1 — The DCE questionnaire. [file 1472-6963-13-454-S1.pdf]

UBC

BC Cancer Agency
CARE & RESEARCH
An agency of the Provincial Health Services Authority

CORE
Collaboration for Outcomes Research and Evaluation

Start

Study of Preferences for Delivery of A Genetic Test for Treatment of a Fast-acting Curable Cancer

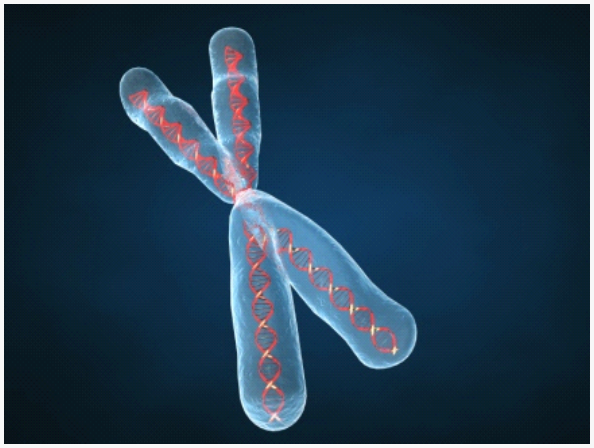

Next

I1

Introduction

Thank you for participating in this survey that we are conducting at the BC Cancer Research Center to measure people's preferences regarding the use of genetic tests in cancer treatment. This study is being sponsored by Genome BC .

I2

About the survey

This survey will take approximately 20 minutes to complete. Your participation is optional, and you may choose to not answer any survey questions and/or withdraw your participation at any time. Your rights to privacy are protected by both federal and provincial laws which ensure that any responses you provide will be kept confidential.

The survey will describe a hypothetical disease scenario. Then you will be asked a series of questions regarding your preferences for a genetic test. This genetic test can help you to decide among your treatment options. Please read the following introduction about the survey questions. You can go back to review or change your answers by clicking back button of your internet browser at any point in the survey.

Next

0% 100%

I3

Curable cancer

Imagine that you have recently been diagnosed with a fast-acting but curable form of cancer. Currently, approximately 50 out of 100 (50%) of patients are cured after the first round of chemotherapy . If you are cured by this initial treatment, you will have a normal life expectancy, otherwise your life expectancy is approximately 1 year. In this case you will be given the secound round of chemotherapy but your chance of being cured is about 10 out of 100 (10%).

I4

The new medication for curable cancer

By adding a new medication to the first round of chemotherapy the cure rate increases from 50 out of 100 (50%) to 75 out of 100 (75%) . However, only some of individuals can benefit from the new medication (responders) and other individuals receive absolutely no benefit from adding the new medication to the standard chemotherapy (non-responders).

The downside of adding the new medication to the standard chemotherapy is that it increases the likelihood and severity of treatment side-effects.

Next

0% 100%

UBC

BC Cancer Agency CARE & RESEARCH An agency of the Provincial Health Services Authority

Collaboration for Outcomes Research and Evaluation

I5

The genetic test associated with the new medication

A new genetic test has just been developed for predicting whether you are a responder or a non-responder to the new medication. If you choose to not take the genetic test, you will receive the new medication in addition to standard chemotherapy without knowing if you are a responder or a non-responder.

I6

The benefits of the genetic test

The advantage of taking the genetic test is that if you learn that you are a non-responder you would recieve the standard chemotherapy without adding the new medication and therefore you could avoid the asscoiated side effects of the new medication.

I7

The downsides of the genetic test

The problem of the genetic test is that sometimes it gives incorrect results:

Some responders will be diagnosed as non-responders. These patients who could be cured by taking the new medication, will not receive it based on the wrong genetic test results.

Also some non-responders will be diagnosed as responders and the new medication will be added to their regular chemotherapy. They will receive absolutely no additional benefit from the new medication. However, they may experience side-effects of the new medication.

Next

0% 100%

A1

Before you start the survey:

There are several characteristics (“Attributes”) of the genetic test and the new medication that may influence your decision for choosing among your treatment options. Below, you can find descriptions of these attributes.

A2

1. Untreated Responders
Proportion of patients who could be cured by the new medication (responders) but will not receive it as a result of wrong genetic test result. This proportion shows how much error is involved in the genetic test results and it is better to be as low as possible.

A4

2. Unnecessary Treatment of Non-responders
Proportion of patients who would not benefit from the new medication (non-responders) but will receive it as a result of wrong genetic test result. These patients will experience the additional side effects of the new medication without getting any benefit from that. This proportion shows how much error is involved in genetic test results and it is better to be as low as possible

A3

3. Severity of Side Effects
The new medication may be associated with side effects such as nausea, hair loss, skin rash and fatigue.
In the questionnaire below, the potential levels of Side Effect Severity are:

- *Severe:* Presence of one or more of the examples listed above. Assume that the treatment and/or test side effects would significantly impact your quality of life and that this impact would be comparable to or worse than living with the disease.
- *Moderate:* Some side effects associated with both the genetic test and the treatment. If there are any side effects, they are less severe than living with the disease.
- *Minimal:* No side effects or minimal side effects associated with both the genetic test and the treatment. If there are any side effects, they are much less severe than living with the disease.

A6

4. Likelihood of Side Effects
The side effects described in Attribute 3 will not necessarily occur for all individuals. Instead, they will occur with a particular percentage chance. For example, if the likelihood of experiencing side effects is 25%, then 25 out of 100 people would be expected to experience side effects, while the remaining 75 people would experience no side effects.

A5

5. Genetic Test Cost
Under the British Columbia Medical Services Plan, you would not be expected to pay for the genetic test and treatment medications. However, we are interested in knowing how highly you value these genetic test results. For the purposes of this exercise, please assume that you would be paying only for the genetic test out-of-pocket.

A7

6. Genetic Test Turnaround Time

The time required to obtain the genetic test results, after the test has been performed.

A8

7. Genetic Test Procedure

Type of the procedure that is needed for doing the genetic test. The genetic test can be a simple procedure such as mouth swab or blood sample or it can involve an invasive procedure such as collecting a liver or bone marrow sample using a needle following a local anesthesia.

Next

0% 100%

Q2

Please answer the following questions about yourself:

1. What is your age?

Q3

2. What is your sex?

Q3_1 Female Q3_2 Male

Q4

3. What is your highest level of education?

Q4_1 Some high school Q4_2 High school Q4_3 College Q4_4 Bachelor degree Q4_5 Master degree Q4_6 Doctorate

Q6

4. How many dependent children do you have?

Q6_1 0 Q6_2 1 Q6_3 2 Q6_4 3 or more

Q8

5. How do you describe your current health?

Q8_1 Excellent Q8_2 Very good Q8_3 Good Q8_4 With some health problems Q8_5 Having serious health problems

Q9

6. Do you know any one among your family or friends who has been diagnosed with cancer?

Q9_1 Yes, very closely Q9_2 Yes Q9_3 No

Q13

7. What is your household's income range?

Q13_1 Less than 25000 a year Q13_2 Between 25000 to 50000 a year Q13_3 Between 50000 to 75000 a year Q13_4 Between 75000 to 100000 Q13_5 Between 100000 to 125000 a year Q13_6 125000 or more

Next

0% 100%

ss

Questionnaire

Imagine that you have been diagnosed with a fast-acting but curable cancer and you should decide whether to receive the new treatment in addition to regular chemotherapy. The genetic test can potentially predict whether you are a responder or a non-responder to the new medication.

Please answer the following questions about your preferences for the new medication and its associated genetic test. In each question you can choose between option A or option B based on their attributes. Also you may prefer not to be tested by choosing the neither option. If you don't take a genetic test, you will be given the new medication in addition to standard chemoterapy without knowing if you are a responder or a non-responder.

Next

0% 100%

CBCRAN1

If these were your only options, which would you choose?
Move the mouse over each attribute for its definition.

Attributes	Option A	Option B	Neither
Untreated Responders	50 out of 100	5 out of 100	0 out of 100
Unnecessary Treatment of Non-responders	50 out of 100	5 out of 100	100 out of 100
Severity of Side Effects	Moderate	Mild	Severe
Likelihood of Side Effects	50 out of 100	5 out of 100	95 out of 100
Cost	$1000	$500	$0
Test Turnaround Time	7 days	2 days	0 days
Genetic Test Procedure	Bone Marrow Biopsy	Blood Sample	None

Next

0% 100%

CBCRAN2

If these were your only options, which would you choose?
Move the mouse over each attribute for its definition.

Attributes	Option A	Option B	Neither
Untreated Responders	35 out of 100	20 out of 100	0 out of 100
Unnecessary Treatment of Non-responders	20 out of 100	35 out of 100	100 out of 100
Severity of Side Effects	Severe	Mild	Severe
Likelihood of Side Effects	95 out of 100	50 out of 100	95 out of 100
Cost	$50	$1500	$0
Test Turnaround Time	12 days	2 days	0 days
Genetic Test Procedure	Tumor Biopsy	Mouth Swab	None

Next

0% 100%

CBCRAN3

If these were your only options, which would you choose?
Move the mouse over each attribute for its definition.

Attributes	Option A	Option B	Neither
Untreated Responders	5 out of 100	20 out of 100	0 out of 100
Unnecessary Treatment of Non-responders	35 out of 100	5 out of 100	100 out of 100
Severity of Side Effects	Moderate	Severe	Severe
Likelihood of Side Effects	95 out of 100	5 out of 100	95 out of 100
Cost	$500	$1000	$0
Test Turnaround Time	7 days	12 days	0 days
Genetic Test Procedure	Tumor Biopsy	Liver Biopsy	None

Next

0% 100%

CBCRAN4

If these were your only options, which would you choose?
Move the mouse over each attribute for its definition.

Attributes	Option A	Option B	Neither
Untreated Responders	35 out of 100	50 out of 100	0 out of 100
Unnecessary Treatment of Non-responders	50 out of 100	20 out of 100	100 out of 100
Severity of Side Effects	Mild	Moderate	Severe
Likelihood of Side Effects	5 out of 100	95 out of 100	95 out of 100
Cost	$50	$1500	$0
Test Turnaround Time	7 days	2 days	0 days
Genetic Test Procedure	Mouth Swab	Liver Biopsy	None

Next

0% 100%

CBCFIX1

If these were your only options, which would you choose?
Move the mouse over each attribute for its definition.

Attributes	Option A	Option B	Neither
Untreated Responders	5 out of 100	20 out of 100	0 out of 100
Unnecessary Treatment of Non-responders	5 out of 100	35 out of 100	100 out of 100
Severity of Side Effects	Mild	Moderate	Severe
Likelihood of Side Effects	5 out of 100	50 out of 100	95 out of 100
Cost	$50	$500	$0
Test Turnaround Time	2 days	7 days	0 days
Genetic Test Procedure	Mouth Swab	Bone Marrow Biopsy	None

Next

0% 100%

CBCRAN5

If these were your only options, which would you choose?
Move the mouse over each attribute for its definition.

Attributes	Option A	Option B	Neither
Untreated Responders	5 out of 100	20 out of 100	0 out of 100
Unnecessary Treatment of Non-responders	50 out of 100	5 out of 100	100 out of 100
Severity of Side Effects	Severe	Mild	Severe
Likelihood of Side Effects	50 out of 100	95 out of 100	95 out of 100
Cost	$1500	$50	$0
Test Turnaround Time	12 days	7 days	0 days
Genetic Test Procedure	Blood Sample	Bone Marrow Biopsy	None

Next

0% 100%

CBCRAN6

If these were your only options, which would you choose?
Move the mouse over each attribute for its definition.

Attributes	Option A	Option B	Neither
Untreated Responders	50 out of 100	35 out of 100	0 out of 100
Unnecessary Treatment of Non-responders	35 out of 100	20 out of 100	100 out of 100
Severity of Side Effects	Moderate	Severe	Severe
Likelihood of Side Effects	5 out of 100	50 out of 100	95 out of 100
Cost	$1000	$500	$0
Test Turnaround Time	12 days	2 days	0 days
Genetic Test Procedure	Mouth Swab	Bone Marrow Biopsy	None

Next

0% 100%

UBC

BC Cancer Agency
CARE & RESEARCH
An agency of the Provincial Health Services Authority

CORE
Collaboration for Outcomes Research and Evaluation

CBCRAN7

If these were your only options, which would you choose?
Move the mouse over each attribute for its definition.

Attributes	Option A	Option B	Neither
Untreated Responders	20 out of 100	50 out of 100	0 out of 100
Unnecessary Treatment of Non-responders	20 out of 100	5 out of 100	100 out of 100
Severity of Side Effects	Moderate	Mild	Severe
Likelihood of Side Effects	5 out of 100	50 out of 100	95 out of 100
Cost	$1500	$500	$0
Test Turnaround Time	7 days	12 days	0 days
Genetic Test Procedure	Blood Sample	Tumor Biopsy	None

Next

0% 100%

CBCRAN8

If these were your only options, which would you choose?
Move the mouse over each attribute for its definition.

Attributes	Option A	Option B	Neither
Untreated Responders	5 out of 100	35 out of 100	0 out of 100
Unnecessary Treatment of Non-responders	50 out of 100	35 out of 100	100 out of 100
Severity of Side Effects	Severe	Moderate	Severe
Likelihood of Side Effects	95 out of 100	50 out of 100	95 out of 100
Cost	$1000	$50	$0
Test Turnaround Time	2 days	12 days	0 days
Genetic Test Procedure	Liver Biopsy	Blood Sample	None

Next

0% 100%

CBCFIX2

If these were your only options, which would you choose?
Move the mouse over each attribute for its definition.

Attributes	Option A	Option B	Neither
Untreated Responders	20 out of 100	5 out of 100	0 out of 100
Unnecessary Treatment of Non-responders	35 out of 100	5 out of 100	100 out of 100
Severity of Side Effects	Moderate	Mild	Severe
Likelihood of Side Effects	50 out of 100	5 out of 100	95 out of 100
Cost	$500	$50	$0
Test Turnaround Time	7 days	2 days	0 days
Genetic Test Procedure	Bone Marrow Biopsy	Mouth Swab	None

Next

0% 100%

CBCRAN9

If these were your only options, which would you choose?
Move the mouse over each attribute for its definition.

Attributes	Option A	Option B	Neither
Untreated Responders	5 out of 100	50 out of 100	0 out of 100
Unnecessary Treatment of Non-responders	20 out of 100	35 out of 100	100 out of 100
Severity of Side Effects	Mild	Severe	Severe
Likelihood of Side Effects	95 out of 100	5 out of 100	95 out of 100
Cost	$1000	$50	$0
Test Turnaround Time	7 days	2 days	0 days
Genetic Test Procedure	Mouth Swab	Tumor Biopsy	None

Next

0% 100%

CBCRAN10

If these were your only options, which would you choose?
Move the mouse over each attribute for its definition.

Attributes	Option A	Option B	Neither
Untreated Responders	20 out of 100	35 out of 100	0 out of 100
Unnecessary Treatment of Non-responders	50 out of 100	5 out of 100	100 out of 100
Severity of Side Effects	Moderate	Severe	Severe
Likelihood of Side Effects	95 out of 100	50 out of 100	95 out of 100
Cost	$500	$1500	$0
Test Turnaround Time	12 days	7 days	0 days
Genetic Test Procedure	Bone Marrow Biopsy	Liver Biopsy	None

Next

0% 100%

CBCRAN11

If these were your only options, which would you choose?
Move the mouse over each attribute for its definition.

Attributes	Option A	Option B	Neither
Untreated Responders	50 out of 100	35 out of 100	0 out of 100
Unnecessary Treatment of Non-responders	35 out of 100	50 out of 100	100 out of 100
Severity of Side Effects	Severe	Mild	Severe
Likelihood of Side Effects	95 out of 100	5 out of 100	95 out of 100
Cost	$500	$1000	$0
Test Turnaround Time	7 days	2 days	0 days
Genetic Test Procedure	Blood Sample	Tumor Biopsy	None

Next

0% 100%

CBCRAN12

If these were your only options, which would you choose?
Move the mouse over each attribute for its definition.

Attributes	Option A	Option B	Neither
Untreated Responders	5 out of 100	20 out of 100	0 out of 100
Unnecessary Treatment of Non-responders	5 out of 100	20 out of 100	100 out of 100
Severity of Side Effects	Moderate	Mild	Severe
Likelihood of Side Effects	50 out of 100	5 out of 100	95 out of 100
Cost	$50	$1500	$0
Test Turnaround Time	2 days	12 days	0 days
Genetic Test Procedure	Mouth Swab	Bone Marrow Biopsy	None

Next

0% 100%

CBCRAN13

If these were your only options, which would you choose?
Move the mouse over each attribute for its definition.

Attributes	Option A	Option B	Neither
Untreated Responders	35 out of 100	50 out of 100	0 out of 100
Unnecessary Treatment of Non-responders	5 out of 100	20 out of 100	100 out of 100
Severity of Side Effects	Moderate	Mild	Severe
Likelihood of Side Effects	95 out of 100	50 out of 100	95 out of 100
Cost	$1000	$50	$0
Test Turnaround Time	2 days	7 days	0 days
Genetic Test Procedure	Blood Sample	Liver Biopsy	None

Next

0% 100%

CBCRAN14

If these were your only options, which would you choose?
Move the mouse over each attribute for its definition.

Attributes	Option A	Option B	Neither
Untreated Responders	20 out of 100	5 out of 100	0 out of 100
Unnecessary Treatment of Non-responders	50 out of 100	35 out of 100	100 out of 100
Severity of Side Effects	Moderate	Severe	Severe
Likelihood of Side Effects	50 out of 100	5 out of 100	95 out of 100
Cost	$500	$1500	$0
Test Turnaround Time	7 days	12 days	0 days
Genetic Test Procedure	Tumor Biopsy	Bone Marrow Biopsy	None

Next

0% 100%

Thank you for completing this survey. Before you exit this survey please share with us your thoughts and comments about any aspect of this study such as clarity of questions, relevance, descriptions and definitions or any other issue. Your comments and feedback are greatly appreciated and will help us to improve the next phases of this study.

Next

0% 100%

fin

Note:

This page has a link on it. When the survey is run this page will be redirected to:

http://surveys.ipsosinteractive.com/wix/p375673781.aspx?l= [SCRIPT] &r= [SCRIPT] &s= [SCRIPT] &BVAR=1&hid_test_mode=1

Powered by Sawtooth Software, Inc.

0% 100%
